# A large field of view 2- and 3-photon microscope

**DOI:** 10.1038/s41377-025-01780-7

**Published:** 2025-02-27

**Authors:** Jack Waters

**Affiliations:** Allen Institute for Brain Science, 615 Westlake Ave N, Seattle, WA 98109 USA

**Keywords:** Imaging and sensing, Microscopy

## Abstract

A new multiphoton fluorescence microscope has been developed, offering cellular resolution across a large field of view deep within biological tissues. This opens new possibilities across a range of biological sciences, particularly within neuroscience where optical approaches can reveal signaling in real time throughout an extended network of cells distributed through the brain of an awake, behaving mouse.

One of the aims of neuroscience is to understand how cells in the brain encode information about the world and drive behaviors. Most sensations and behaviors engage spatially distributed networks of cells^1,2^. An adult mouse brain is large enough, ~1.5 × 1 × 0.5 cm, that multiple electrodes can be implanted to simultaneously measure the activities of tens of thousands of neurons^3,4^, but electrodes are invasive and provide little information on the spatial organization of cells. Fluorescence microscopy provides a complementary approach, imaging ion-sensitive molecules to report activity^5^, and supplying rich spatial information on the underlying cell populations and brain structures.

For many fluorescent ion sensors, excitation and emission wavelengths are in the visible spectrum, where brain tissue is turbid and the mean free path length is ~0.1 mm. 2-photon excitation with near-infrared wavelengths extends the depth limit^6,7^ providing optical access to the superficial ~0.5 mm of tissue. The surface structure of the mammalian brain is neocortex, where sensory information is processed and motor actions planned. Many studies using 2-photon fluorescence microscopy have probed the activities of neocortical cells during the processing of sensory information and behaviors, particularly in the mouse^8^.

Neocortex is arranged in repeating subnetworks, columnar in shape, ~0.5 mm in diameter and extending through the depth of neocortex. Monitoring the interactions between neighboring neocortical subnetworks requires a microscope with a field of view of at least a millimeter so large field of view 2-photon microscopes have been developed, with custom objectives^9^ and often custom scanning and collection optics^10–14^. With 2-photon excitation, it’s possible to monitor the activities of a million cells distributed through ~6 × 6 × 0.5 mm of the brain of an awake, behaving mouse^15^.

Neocortex is 0.7–2 mm deep so 2-photon excitation provides access to only part of each neocortical subnetwork. With 2-photon excitation, imaging depth is limited by fluorescence generated outside the focal plane, a phenomenon that declines with higher-order non-linear excitation^16–19^. In the last few years, lasers suitable for 3-photon excitation have become commercially available (with <50 fs pulse durations, µJ pulse energies, and MHz repetition rates) enabling imaging to 1.5–2 mm, but a large field of view 3-photon microscope capable of imaging several neocortical subnetworks was missing.

In the issue of *eLight*, Aaron Mok and colleagues at Cornell University demonstrate a 3-photon microscope with a ~ 3 × 3 mm field of view^20^. Excitation efficiency was optimized by matching the point spread function to the size of neocortical cells and shuttering the beam between cells, with remote focusing to enable near-simultaneous imaging of cells at different depths. This new microscope provides optical access to neighboring networks of cells throughout the depth of mammalian neocortex, even in large mammals, opening new opportunities to study the cellular basis of mammalian behaviors (Fig. 1).

**Fig. 1:**
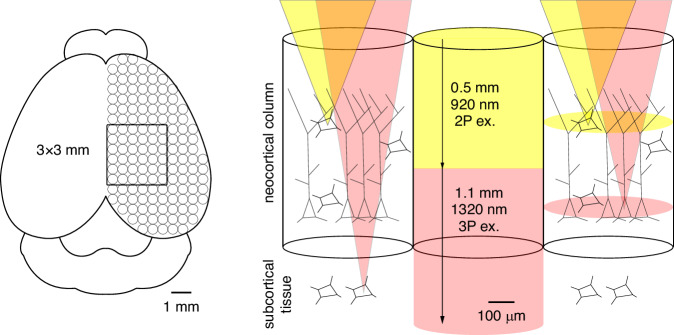
Schematic illustration of a large field of view 2- and 3-photon microscope. Left, top view of a mouse brain showing the approximate size of subnetworks, or columns in neocortex and the 3 mm field of view of the large field of view microscope. Right, the large field of view microscope can image simultaneously from several locations in neighboring neocortical columns and the underlying neocortical tissue using a combination of 2- and 3-photon excitation

The microscope of Mok et al. outperforms 2-photon microscopes in penetration depth, but 2-photon excitation still leads 3-photon excitation in many respects. For example, the highest performance 2-photon microscopes are capable of imaging several orders of magnitude more cells per unit time. A difference is likely to persist since the 2- and 3-photon cross-sections of ion-sensitive fluorophores favor 2-photon excitation^21^, but as higher repetition rate 3-photon lasers become available^22^ the advantage of 2-photon excitation in cells per unit time or, equivalently, samples per unit time per cell^23^ may narrow. Synchronous measurement and manipulation of activity is a second capability where 2-photon excitation dominates^24–28^. We can anticipate 3-photon microscopy developing in these directions, resulting in the capability to measure and manipulate populations of cells deep within the brain, testing causal links between cellular activity and behaviors.
